# Perfil Clínico e Resultados em 30 dias de Pacientes com Válvula Aórtica Bicúspide Submetidos à Válvula Aórtica e/ou Cirurgia da Aorta

**DOI:** 10.36660/abc.20211018

**Published:** 2022-03-10

**Authors:** Paulo Roberto B. Evora

**Affiliations:** 1 Departamento de Cirurgia e Anatomia Faculdade de Medicina de Ribeirão Preto Universidade de São Paulo São Paulo SP Brasil Departamento de Cirurgia e Anatomia, Faculdade de Medicina de Ribeirão Preto, Universidade de São Paulo, São Paulo, SP – Brasil

**Keywords:** Valva Aórtica Bicúspide

Este minieditorial foi motivado pelos resultados apresentados e discutidos em excelente artigo realizado no INCOR,^1^ ressaltando que faltam estudos sobre o perfil desses pacientes na população brasileira. Isso reforça a relevância do estudo em questão, uma vez que a válvula aórtica bicúspide (VAB) afeta 0,5% a 2% das pessoas e está associada a alterações valvares e aórticas.

A VAB é uma alteração na embriogênese da aorta, válvula que não é totalmente compreendida ainda que se tenha várias teorias sobre sua origem. Essas teorias incluem:^2 , 3^

1. A alteração no fluxo transvalvar fetal leva à falha na separação das cúspides.
2. Fatores genéticos.
3. Falha na migração celular em alguns estágios da embriogênese.

A rigidez arterial é um preditor essencial de aortopatia e remodelação miocárdica em 41 pacientes com VAB, e pode estar aumentada na infância. Por esse motivo, há um interesse crescente no acompanhamento de pacientes com VAB desde a infância. Um artigo recente de Pelin Kosger et al.,^4^ publicado nos Arquivos Brasileiros de Cardiologia, avalia a rigidez arterial e a função miocárdica do ventrículo esquerdo em 44 crianças com uma VAB funcionando bem. A investigação revelou que, de acordo com a análise da onda de pulso oscilométrica de crianças com VAB em bom funcionamento, apresentavam rigidez arterial semelhante à de seus pares saudáveis. O diâmetro da aorta ascendente foi estabelecido como um preditor independentemente da função miocárdica do ventrículo esquerdo. Portanto, a rigidez arterial pode não ser um fator de risco grave em pacientes pediátricos sem dilatação acentuada da aorta ascendente.^4^

O estudo do INCOR é uma coorte retrospectiva incluindo 195 pacientes (idade média 54 ± 14 anos, 73,8% do sexo masculino) com diagnóstico de VAB submetidos à abordagem cirúrgica (valvular e aorta) de 2014 a 2019. Foram avaliados dados clínicos, ecocardiográficos e tomográficos, além das características da intervenção e evolução em 30 dias. Os resultados revelaram:

1. Alta prevalência de aneurisma de aorta (56,5%), com diâmetro médio de 46,9 ± 10,2 mm.
2. Regurgitação aórtica importante em 25,1% e estenose aórtica significativa em 54,9%. A cirurgia isolada da valva aórtica foi realizada em 48,2%, a cirurgia isolada da aorta em 6,7% e a cirurgia combinada em 45,1%. A mortalidade em 30 dias foi de 8,2%.

Na análise multivariada, os preditores do desfecho combinado em 30 dias (óbito, fibrilação atrial e reoperação) foram: idade (OR 1,044, IC 95% 1,009-1,081, p = 0,014) e índice de massa ventricular esquerda (OR 1,009,995 % CI 1,000-1,018, p = 0,044). Talvez o achado mais crítico seja que os pacientes com VAB apresentam maior incidência de aortopatia, com a necessidade adicional de avaliação da aorta com tomografia computadorizada ou ressonância magnética. Outro fato que merece destaque foi a discussão de que, em geral, as características da população brasileira não apresentam diferenças marcantes em relação aos dados de outros países, e a organização das diretrizes internacionais é mais coerente. Resta a infindável velha história da incidência da febre reumática, diferenciada de acordo com o nível de desenvolvimento das populações.

Os autores ressaltam que a principal limitação deste estudo é inerente ao seu desenho observacional. Assim, dados que pudessem influenciar negativamente o resultado cirúrgico e eventos de impacto (como tempo de circulação extracorpórea, internação hospitalar, uso de drogas vasoativas, uso de suporte circulatório, taxa de infecção entre outros) não estavam disponíveis para análise em todos os pacientes. Além disso, o seguimento de curto prazo (30 meses) não permite extrapolar os achados para além desse período. Aguardamos as avaliações dos resultados de médio e longo prazo.

Atualmente, não existem tratamentos específicos conhecidos para evitar que a válvula bicúspide desenvolva estenose ou regurgitação. As estatinas para reduzir o colesterol podem ajudar algumas pessoas. No entanto, minha dúvida sobre o assunto, que acredito ser compartilhada pela maioria dos cirurgiões cardíacos, surgiu na evidência de que as VAB estão sempre associadas com dilatações aórticas. Justifica-se associar a abordagem cirúrgica da aorta em todos os casos de VAB? As grandes diretrizes não apresentam uma abordagem definitiva, podendo ser assumidas como um comportamento lógico tendencial, mas difíceis de enquadrar nos princípios de Hipócrates “mutilar ao mínimo, reconstruir ao máximo ...”
