# Supplementary material for: Perfil Clínico e Resultados em 30 dias de Pacientes com Válvula Aórtica Bicúspide Submetidos à Válvula Aórtica e/ou Cirurgia da Aorta
Source: Arq Bras Cardiol. 2022 Mar 10;118(3):597–8. [Article in Portuguese] doi: 10.36660/abc.20211018 (PMC8959031; doi:10.36660/abc.20211018)
Supplement: Translation

# Translation: Clinical Profile and Outcomes in 30 Days of Patients with Bicuspid Aortic Valve Undergoing Aortic Valve and/or Aortic Surgery

**Authors:** Paulo Roberto B. Evora

**Affiliations:** 1 Brazil Departamento de Cirurgia e Anatomia, Faculdade de Medicina de Ribeirão Preto, Universidade de São Paulo, São Paulo, SP – Brazil

**Keywords:** Bicuspid Aortic Valve

This short editorial is motivated by the results presented and discussed in an excellent article carried out at INCOR.^1^ The authors emphasize a lack of Brazilian population, strengthening the relevance question since the bicuspid aortic valve (BAV) affects 0.5 to 2% of people and is associated with valve and aortic changes. The bicuspid aortic valve (BAV) is defective embryogenesis of the aortic valve that is not fully understood, even several theories about its origin. These theories include:^2 , 3^

1. Alteration in fetal transvalvular flow leading to failure in cusp separation;
2. Genetic factors;
3. Cell migration failure in some stages of embryogenesis.

Arterial stiffness is an essential predictor of aortopathy and myocardial remodeling in 41 patients with a bicuspid aortic valve, and it might be increased in childhood. For this reason, there has been growing interest in the follow-up of patients with ABV since childhood. A recent article was published in the Brazilian Archives of Cardiology, a very well-written article by Pelin Kosger et al.^4^ To assess arterial stiffness and left ventricular myocardial function in forty-four children with a well-functioning bicuspid aortic valve. The investigation revealed that According to the oscillometric pulse wave analysis, the children with a well-functioning bicuspid aorta valve had similar arterial stiffness to that of their healthy peers. The ascending aorta diameter was established as an independent predictor of left ventricular myocardial function. Arterial stiffness may not be a severe risk factor in pediatric patients without marked ascending aorta dilation.^4^

The INCOR study is a retrospective cohort including 195 patients (mean age 54±14 years, 73.8% male) with a diagnosis of BAV who underwent surgical approach (valvular and aorta) from 2014 to 2019. Clinical echocardiography and tomography data were evaluated, in addition to the characteristics of the intervention and events within 30 days. The results revealed:

1. High prevalence of aortic aneurysm (56.5%), with a mean diameter of 46.9±10.2 mm;
2. Major aortic regurgitation in 25.1% and significant aortic stenosis in 54.9%. Isolated aortic valve surgery was performed in 48.2%, isolated aortic surgery in 6.7%, and combined surgery in 45.1%. The 30-day mortality was 8.2%.

In the multivariate analysis, the predictors of the combined outcome at 30 days (death, atrial fibrillation, and reoperation) were age (OR 1.044, 95% CI 1.009-1.081, p=0.014) and left ventricular mass index (OR 1.009, 95% CI 1.000-1.018, p=0.044). Perhaps the most critical finding was that patients with BAV have a higher incidence of aortopathy, with the additional need to assess the aorta with computed tomography or magnetic resonance imaging. Another fact that deserves to be highlighted was the discussion that, in general, the characteristics of the Brazilian population do not present marked differences compared to data from other countries, and the organization of international guidelines is more coherent. There remains the endless old story of the incidence of Rheumatic Fever, differentiated according to the social level of country populations development.

The authors point out that the main limitation of this study is inherent to its observational design. Thus, data that could negatively influence the surgical outcome and impact events (such as cardiopulmonary bypass time, hospital stay, use of vasoactive drugs, circulatory support, infection rate, among others) were not available for analysis in all patients. Furthermore, the short-term follow-up (30 months) does not allow the findings to be extrapolated beyond this period. We wait for the middle and long time outcomes evaluations.

Currently, no treatments prevent the bicuspid valve from developing stenosis or regurgitation. Statins to lower cholesterol may help some people. However, my capital doubt is, which I believe shared by most cardiac surgeons, arose in the evidence that bicuspid aortic valves are associated with aortic dilations. Is it justified to associate the surgical approach to the aorta in all cases of bicuspid aortic valves? The guidelines do not present a definitive direction, and it can be assumed as a tendential logical behavior, but difficult to fit into the principles of Hippocrates (”mutilate to the minimum, rebuild to the maximum...”)
